# Radiobiological evaluation of the therapeutic effect of silver-111 for the ISOLPHARM project

**DOI:** 10.3389/fnume.2026.1773638

**Published:** 2026-03-12

**Authors:** Alberto Arzenton, Aurora Leso, Francesca Rana, Giulia S. Valli, Elena Delgrosso, Laura Cansolino, Cinzia Ferrari, Silva Bortolussi, Isabella Guardamagna, Giorgio Baiocco, Giorgio Grosso, Davide Serafini, Andrea Gandini, Valerio Di Marco, Antonietta Donzella, Emilio Mariotti, Marcello Lunardon, Devid Maniglio, Germano Bonomi, Alberto Andrighetto

**Affiliations:** 1 Department of Physics and Astronomy “G. Galilei,” University of Padova, Padova, Italy; 2 Padova Division, National Institute for Nuclear Physics (INFN), Padova, Italy; 3 Legnaro National Laboratories, INFN, Legnaro, Italy; 4 Department of Physics and Earth Sciences, University of Ferrara, Ferrara, Italy; 5 Department of Physics “Alessandro Volta,” University of Pavia, Pavia, Italy; 6 Department of Physical Sciences, Earth and Environment, University of Siena, Siena, Italy; 7 Department of Civil Engineering and Architecture, University of Pavia, Pavia, Italy; 8 Department of Clinical Surgical Sciences, Integrated Unit of Experimental Surgery, Advanced Microsurgery and Regenerative Medicine, University of Pavia, Pavia, Italy; 9 Pavia Division, INFN, Pavia, Italy; 10 Department of Chemical Sciences, University of Padova, Padova, Italy; 11 Applied Nuclear Energy Laboratory (LENA), University of Pavia, Pavia, Italy; 12 Department of Industrial and Mechanical Engineering, University of Brescia, Brescia, Italy; 13 Pisa Division, INFN, Pisa, Italy; 14 Trento Institute for Fundamental Physics and Applications, INFN, Trento, Italy; 15 Department of Industrial Engineering, University of Trento, Biotech Center for Biomedical Technologies, Trento, Italy

**Keywords:** clonogenic survival, *foci*, ISOLPHARM, linear quadratic model, micronuclei, radiobiology, silver-111, targeted radionuclide therapy

## Abstract

**Introduction:**

The ISOLPHARM project has the aim of developing novel radiopharmaceuticals using the wide choice of radionuclides produced by Isotope Separation OnLine (ISOL) at LNL-INFN in the SPES facility, which is currently nearing completion. One of the most promising candidates for Targeted Radionuclide Therapy (TRT) is the beta-emitting radiometal silver-111, obtainable carrier-free irradiating a uranium carbide target with a proton beam and applying the ISOL technique. Until SPES will become fully operational, small quantities of silver-111 are produced by the TRIGA Mark II nuclear reactor hosted by the LENA facility of the University of Pavia to begin the preclinical research. The present work concerns the first radiobiological experiment involving silver-111.

**Methods:**

Different activity concentrations of the aforementioned radiometal are administered to the UMR-106 rat osteosarcoma and LNCaP human prostate cancer cell lines through the culture medium. The survival curves after four and six days of exposure, as well as the recurrence of *foci* of DNA repair proteins and micronuclei, are evaluated as a function of the absorbed dose and compared to the control cultures. According to the MIRD formalism, a dosimetric analysis is performed taking advantage of cellular S-values simulated with the Monte Carlo code Geant4 in a generalized cell geometry. This makes it possible to relate the experimental outcome, namely the surviving cells after the exposure cycles, to the absorbed dose in the cell nucleus or in the whole cell environment.

**Results and discussion:**

The results show a difference in the response of the two cell lines, probably due to the thresholds of their DNA repair pathways, and highlight a possible weakness of the linear-quadratic model when applied to this kind of radiobiological studies.

## 1 Introduction

The continuous growth of nuclear medicine at the present day is pivotal in the fight against cancer, thanks to the development and refinement of several cutting-edge techniques for diagnosis and therapy [1, 2]. Radionuclides with different properties can be used by physicians to adopt personalized approaches tailored to each specific patient. In this scenario, radiometals, whose chemical behavior is in many cases suitable for the synthesis of targeted macromolecules, are of increasing importance in α or $\beta^{-}$ Targeted Radionuclide Therapy (TRT). Concerning $\beta^{-}$ emitters, a relevant example is given by drugs based on lutetium-177, officially approved for the first time by the FDA and EMA in 2022 for the treatment of prostatic tumors [3]. Other common $\beta^{-}$ emitters in nuclear medicine are strontium-89, yttrium-90, iodine-131 and samarium-153: their half-lives, ranging from 2 to 8 d (with the exception of 50 days for strontium-89), are considered optimal for therapeutic applications [4].

Besides these $\beta^{-}$ emitters, several other candidates are currently being studied. This is the case of rhenium-186, being tested against prostatic cancers in phase I/II clinical trials [5], and of silver-111 (^111^Ag), whose decay products closely resembles that of the former nuclide. The half-life of ^111^Ag is 7.45 d and its most intense $\beta^{-}$ emission has an average energy of 360.4 keV (89%); moreover, it decays into its stable daughter nucleus cadmium-111 emitting low-energy γ rays (the most intense is 6.7% at 342.13 keV), which could be imaged by SPECT during or after therapy, as can be done with lutetium-177 [3, 6]. However, although its decay radiation has optimal properties for therapy, the use of expensive palladium-110 targets required for routine ^111^Ag production in nuclear reactors [7–9], as well as the non-trivial chelation of silver, has hindered its medical application until now.

In this context, a new perspective on the medical application of ^111^Ag has been provided by the ISOLPHARM project, whose aim is to develop innovative radiopharmaceuticals using the nuclides produced via Isotope Separation OnLine (ISOL) in the Selective Production of Exotic Species (SPES) facility, which is currently under commissioning at the Legnaro National Laboratories of the National Institute for Nuclear Physics (LNL-INFN) [10, 11]. Carrier-free ^111^Ag will be produced at SPES with high purity and in quantities suitable for clinical applications [12]. For this reason, an experimental campaign to test new compounds labeled with this radionuclide, its therapeutic properties and its imaging potential started in 2018 and is ongoing. Important achievements include improvements in the stability of chelators for silver [13], the synthesis of new targeting molecules [14], the routine production of preclinical quantities of ^111^Ag in the TRIGA Mark II nuclear research reactor at the Applied Nuclear Energy Laboratory (LENA) of the Pavia University [7] and the subsequent first *in-vivo* and *in-vitro* experiments.

The present work focuses on the first *in-vitro* experiments involving free ^111^Ag, performed in a radiobiology laboratory located in the Experimental Surgery Unit of the Department of Clinical-Surgical, Diagnostic and Pediatric Sciences of the Pavia University. The cell lines selected for the first tests are:

1. UMR-106 rat osteosarcoma, with the aim of observing the response of an easy-to-grow cancer cell line to the decay radiation of ^111^Ag administered in different amounts. This line is well-known in our laboratory due to previous experiments with different external radiation beams [15].
2. LNCaP human prostate cancer, to obtain important data with unbound ^111^Ag that may be compared, in the future, to labeled compounds targeted towards the Prostate-Specific Membrane Antigen (PSMA).

To this end, the clonogenic, *foci* of DNA repair proteins and micronuclei assays were chosen as the most meaningful indicators of radiation effects; the protocols are described in Section 2 together with the production and chemical separation procedure and the Monte Carlo simulations for cell dosimetry. The experimental results are shown in Section 3 and finally discussed in Section 4.

## 2 Materials and methods

### 2.1 Radionuclide production and separation

^111^Ag is produced in the nuclear research reactor TRIGA Mark II at LENA (Pavia, Italy) from ^110^Pd-enriched samples with the reaction:

Pd110(n,γ)Pd111→[t1/2=23.4min]β−Ag110.

The usage of enriched ^110^Pd target instead of its natural form is motivated by the fact that it significantly improves ^111^Ag production while at the same time reducing the formation of contaminants. Indeed, using natural Pd leads to the production of several undesired nuclides, both stable and radioactive, including silver isotopes that cannot be chemically separated. It is important to note that even by using an enriched target, traces of metal impurities are still present. Therefore, chemical separation is required, also to enable the recovery of the valuable target material. As a matter of fact, given that ^110^Pd is an expensive material, implementing an efficient recycling strategy for its recovery is essential for the sustainability of future productions.

The obtained irradiated target is first dissolved in aqua regia and then treated with 12 M HCl. Then, the solution is dissolved in 0.005 M HCl with added NaCl and treated with 800 mg of LN resin (Triskem). Finally, a conditioning for separation is performed with 0.005 M HCl. Palladium is extracted and its complete removal can be confirmed by a color change in the solution. Silver is eluted using 1 M HCl.

After the separation, the fractions containing palladium are treated in order to recover as much target material as possible. To do so, the solvent is evaporated and the resulting solid is treated with 0.1 M HCl and 20% NaBH_4_, stirred for one hour, and finally transferred to a 50 mL centrifuge flask. Palladium is precipitated by adding concentrated HCl overnight, followed by centrifugation that allows to remove the supernatant. The material is then washed three times with distilled water at first, ethanol as second step and diethyl ether as last. The recovered solid is left to dry overnight in a vial [7].

In the final physiological solution administered to the cell cultures, the inertness of ^111^Ag is achieved by adding ${\mathrm{Cl}}^{-}$ ions in a much higher concentration. Considering the amounts of ${[}^{111}\mathrm{Ag}]{\mathrm{Ag}}^{+}$ and ${\mathrm{Cl}}^{-}$, together with the volumes employed, the chemical composition of the solutions administered to the cells is as follows: ${[}^{111}\mathrm{Ag}]{\mathrm{Ag}}^{+}$ $1.23\times 10^{-7}$ M and ${\mathrm{Cl}}^{-}$ $2.46\times 10^{-1}$ M. Chemical computations aimed at determining the speciation of this solution can be performed. Given that ${\mathrm{Ag}}^{+}$ and ${\mathrm{Cl}}^{-}$ can form an insoluble salt, AgCl(s) ($K_{s}=1.77\times 10^{-10}$), and two soluble complexes, AgCl (overall formation constant $\beta =2\times 10^{3}$) and ${\mathrm{AgCl}}_{2}^{-}$ ($\beta =1.86\times 10^{5}$), calculations reveal that the solutions administered to the cells contain mainly the soluble complex ${[}^{111}\mathrm{Ag}]{\mathrm{AgCl}}_{2}^{-}$ (96%) and, to a minor extent, the soluble complex ${[}^{111}\mathrm{Ag}]$AgCl (4%), and that no free ${\mathrm{Ag}}^{+}$ and no AgCl(s) are present.

The toxicity of stable solutions of Ag, Pd and Ag-Pd mixtures on cell cultures was investigated in a previous study [16] by means of clonogenic assays, also to evaluate the possible implications of Pd separation residuals. In each of these cases, the clonogenic survival did not show any correlation with the increase of administered concentration; hence, it was concluded that chemical toxicity is negligible compared to radiation-induced effects.

### 2.2 Cell line cultures

The rat osteosarcoma UMR-106 cell line is obtained from the European Collection of Cell Cultures (ECACC). Cells are cultured as monolayers in 75 cm^2^ filter flasks at 37 ^∘^C in a humidified atmosphere with 5% CO_2_. The culture medium consists of high-glucose Dulbecco’s Modified Eagle Medium (DMEM; Euroclone, Italy), supplemented with 10% fetal bovine serum and 40 μg/mL gentamicin. Experiments are also carried out with the human cell line of prostate carcinoma LNCaP. Cells grow adherent in RPMI 1,640 culture medium enriched with 10% of bovine fetal serum and 1% of pen/strep. The tested activity concentrations, chosen with the method explained below, were: 59, 119, 239, and 478 kBq/mL at 4 d of exposure and 43, 87, 174, and 347 kBq/mL at 6 d of exposure. At the end of each exposure period (4 or 6 d), the silver-enriched culture medium is removed and the Petri dishes are processed for the clonogenic assay. The passage numbers are comparable for both cell lines and, in any case, below 20.

### 2.3 Cell dosimetry

In the context of TRT, it is crucial to consider dosimetry at the cell level in order to correctly evaluate the effectiveness of radiopharmaceuticals. To address these needs, the Medical Internal Radiation Dose (MIRD) Committee of the American Society of Nuclear Medicine & Molecular Imaging (SNMMI) has played a central role by developing an algorithm to calculate the radiation dose in systems with different geometries exposed to radioactivity distributions [17]. According to the MIRD schema, a system is considered as a collection of volumes: the radioactive ones are considered as *source* volumes, while the volumes in which the dosimetry is computed are identified as *target* volumes. A single volume can be considered simultaneously target and source. Under the assumption of fixed shape and mass for each volume, the dose rate at time t in a target region $r_{t}$ can be expressed as

$$\dot{D}(r_{t},t)=\sum_{s}A(r_{s},t)\;S(r_{t}\leftarrow r_{s}),$$ (1)

where $A(r_{s},t)$ is the activity in the source volume at time t and $S(r_{t}\leftarrow r_{s})$ is the so-called S-value, which represents the mean absorbed dose per unit of cumulated activity. The latter can be defined as

$$S(r_{t}\leftarrow r_{s})=\frac{1}{m_{t}}\sum_{i}E_{i}Y_{i}\phi (r_{t}\leftarrow r_{s},E_{i}),$$

where $m_{t}$ is the mass of the target region, $E_{i}$ is the mean energy of the i-th decay transition, $Y_{i}$ is the branching ratio and $\phi (r_{t}\leftarrow r_{s},E_{i})$ represents the average fraction of energy $E_{i}$ transferred from $r_{s}$ to $r_{t}$. In order to consider the total number of decays over the exposure time T, the cumulative activity is needed and can be computed as:

$$\tilde{A}(r_{s},T)=\int_{0}^{T}A(r_{s},t)\;dt.$$

By replacing $A(r_{s},t)$ with $\tilde{A}(r_{s},T)$ in Equation 1, the expression for the total absorbed dose over the period T is obtained:

$$D(r_{t},T)=\sum_{s}\tilde{A}(r_{s},T)S(r_{t}\leftarrow r_{s}).$$

In the present work, the radionuclide is not bound to any targeting agent, so we reasonably assume that it is uniformly distributed in the culture medium. Therefore, in order to apply the described formalism in the planning of the *in-vitro* experiments, the dose absorbed by the cell nucleus from the radionuclide in the culture medium in a time window T can be expressed as

$$D(T)=\int_{0}^{T}\dot{D}(t)\;dt=S\cdot A_{c}\cdot \frac{1-e^{-\lambda T}}{\lambda },$$ (2)

where $A_{c}$ represents the initial activity concentration and $\dot{D}(t)$ is the dose rate per cell, which is given by

$$\dot{D}(t)=S\cdot A_{c}\cdot e^{-\lambda t}.$$

The activity concentration required to deliver the desired absorbed dose to a cell can be computed by inverting Equation 1. In this study, $A_{c}$ is known from the radioactivity measurement performed at LENA during the characterization of the sample, while S is taken from a previous work [18]. In particular, the reference reports an S-value around 0.06 mGy/(Bq s) $\mu m^{3}$ for a target cell surrounded by a radioactive medium sphere with the Monte Carlo toolkit Geant4. The radius of this sphere, 3 mm, was found to be a good approximation for a point-like target immersed in a ^111^Ag distribution. Here, half of this value is used because in the experiments the cells lie on the bottom of the dish. Finally, in relation to *T*, the two values of 4 and 6 d were chosen to observe the effects of different exposure times, namely different dose rates for each absorbed dose.

A possible internalization of ^111^Ag in the cell cytoplasm, where it was observed that silver ions can accumulate in the lysosomal fraction or bind to metallothioneins [19, 20], can also be evaluated. To do this, the impact of an equal concentration of intracellular and extracellular ^111^Ag with respect to the non-internalizing case can be studied using the S-values and the cell volumes reported in the same cell dosimetry reference [18]. In particular, the value of 0.075 mGy/(Bq s) for the dose absorbed by an ellipsoidal cell nucleus exposed to a uniform radioactive source of ^111^Ag in the cytoplasm, obtained with Geant4, can be used.^1^ However, the absorbed dose calculated under this hypothesis differs from the previous situation by less than 1%, meaning that this effect is negligible under the experimental conditions adopted. Finally, according to the toxicity test with stable Ag described above, significant biological effects of such internalization mechanisms are excluded.

### 2.4 Clonogenic assay

The clonogenic assay, first introduced by Puck and Marcus in 1955 [21], is a well-established technique in radiation biology used to assess the ability of a single cell to maintain its reproductive integrity after exposure to damaging agents. This method remains the gold standard for evaluating cellular radiosensitivity and long-term survival [22, 23]. Although time-consuming, it is able to provide highly reliable data on cytotoxicity and proliferative capacity. In this study, cells are exposed to increasing doses of ^111^Ag while still adherent to the culture plates, a method commonly adopted to maintain cellular integrity during treatment [24, 25]. 10,000 UMR-106 and 50,000 LNCaP cells are seeded in 35 mm Petri dishes with 3 mL of culture medium, in triplicate. After exposure, cells are detached via trypsinization, counted using a Bürker chamber and seeded at various densities (50–1,000 cells per 60 mm Petri dish), depending on the expected survival rate. For each condition, three replicates are prepared to ensure statistical robustness. Cells are incubated under standard culture conditions (37 ^∘^C, 5% CO_2_) for 8 d to allow for colony formation. The culture medium is replaced after 5 d. At the end of the incubation period, colonies are gently washed with Hank’s buffered saline (Euroclone, Italy), fixed with 70% ethanol and stained with Toluidine Blue. Colonies consisting of at least 50 cells, the minimum number of cells proving that the clone originates from a cell survived to the treatment, are counted under a stereomicroscope. Plating efficiency (PE) is calculated as the ratio of the number of colonies formed to the number of cells seeded. The surviving fraction (SF) is expressed as the PE of treated samples relative to untreated controls. Cell survival curves are then plotted against the absorbed dose.

### 2.5 Foci and micronuclei assay

Cells are seeded on sterile 24 × 24 mm glass coverslips (3,000 cells/well for the UMR cell line and 50,000 cells/well for the LNCaP cell line) in 6-well plates, 48 h prior to ^111^Ag exposure. After 4 or 6 d of treatment, the medium is removed and cells are fixed with 4% paraformaldehyde for 15 min at room temperature, washed with PBS, permeabilized (0.25% Triton X-100 in PBS) and blocked (10% goat serum, 1% BSA, 0.3 M glycine, 0.1% Tween-20 in PBS) for 1 h at 37 ^∘^C. Coverslips are incubated with primary antibodies against γ-H2AX (Millipore, 05-636) and 53BP1 (Abcam, ab21083) diluted 1:1000 in blocking buffer for 1 h at 37 ^∘^C or overnight at 4 ^∘^C. After washes with a buffer composed by 0.2% Triton X-100 in PBS, cells are incubated for 1 h at 37 ^∘^C in the dark with Alexa Fluor 555 anti-mouse and Alexa Fluor 488 anti-rabbit secondary antibodies (1:1000 in blocking buffer). Additional washes are performed to remove excess of secondary antibodies and coverslips are mounted with ProLong Gold Antifade with DAPI and stored overnight at room temperature in the dark. Slides are imaged using a Leica Thunder Live Cell Imager DMi8 fluorescence microscope with a 40× objective, and γ-H2AX and 53BP1 *foci* per nucleus are counted. In addition, DAPI images can also be used for a basic micronuclei assessment.

## 3 Results

This section presents the experimental results obtained by means of clonogenic survival, *foci* and micronuclei assays performed on UMR-106 and LNCaP cells as described in Section 2.

### 3.1 Clonogenic survival

**Figure 1:**
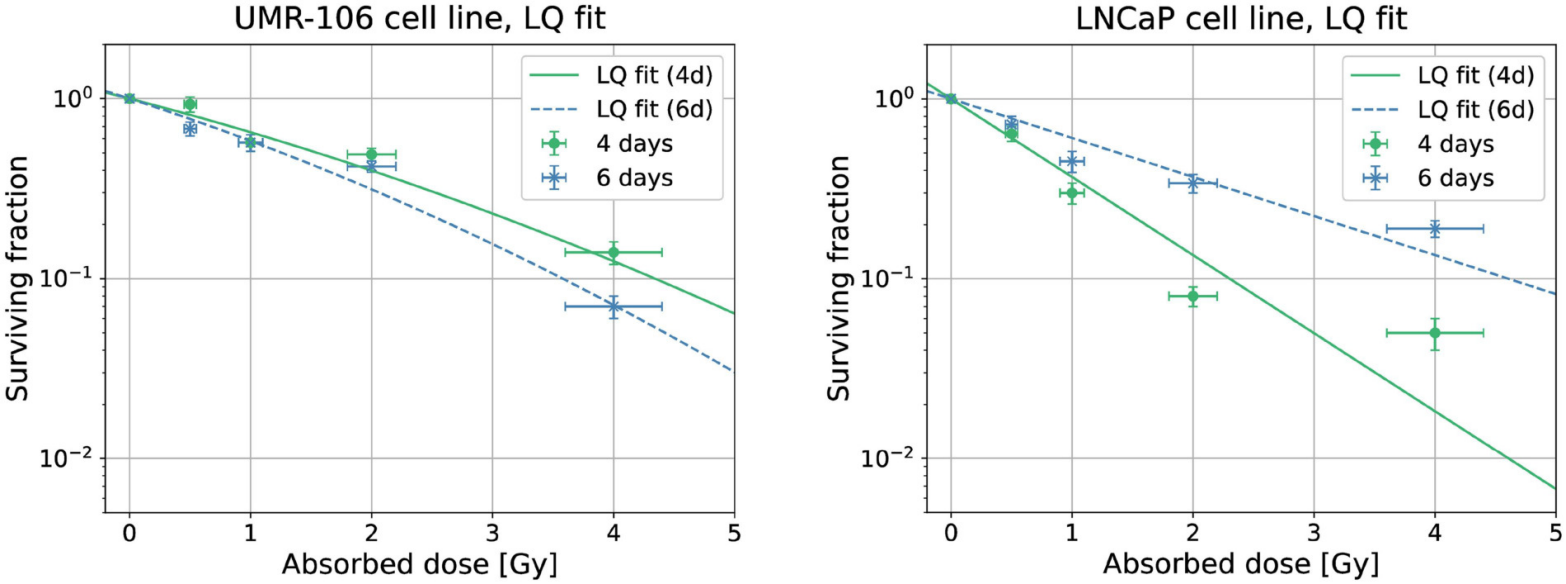
Linear-quadratic fit of the clonogenic survival measured with the UMR-106 (left) and LNCaP (right) cell lines after 4 and 6 d of irradiation.

The clonogenic survival results are shown in Figure 1. The absorbed doses were arbitrarily chosen and, depending on the exposure time, the corresponding activity concentrations were calculated using Equation 2. One can immediately notice that, while for the LNCaP cell line the shorter exposure (and thus the higher dose rate) seems more effective, for the UMR-106 cell line a smaller SF is implied by the longer exposure (lower dose rate). A possible hypothesis is that, with the lower dose rate, certain DNA Double Strand Break (DSB) repair pathways are still not activated by UMR-106 cells, resulting in a higher death probability. In contrast, LNCaP cells do not appear to be below any particular repair threshold, but rather their repair mechanisms seem to work better when the dose rate is lower and, therefore, the damage yield is easier to repair. The influence of repair dynamics on the outcome of this study is also suggested by the Linear-Quadratic (LQ) fit of the data. Looking at the fit parameters, in Table 1, it can be seen that the quadratic parameter β is always compatible with zero or even estimated exactly as zero in the case of LNCaP cells. The model is then reduced to only its linear component, regulated by the parameter α. This behavior, common with high-LET radiation (such as α-ray emission), may mean that the $\beta^{-}$ particles of ^111^Ag caused complex, hardly reparable DNA damage to the studied cell lines.

**Table 1:** Linear-quadratic fit parameters of the clonogenic survival measured with the UMR-106 and LNCaP cell lines after 4 and 6 d of exposure to ^111^Ag.

Cell line	Exposure	α [Gy^−1^]	β [Gy^−2^]
UMR-106	4 d	0.4 ± 0.1	0.03 ± 0.04
	6 d	0.5 ± 0.1	0.04 ± 0.05
LNCaP	4 d	1.0 ± 0.8	0.00 ± 0.01
	6 d	0.50 ± 0.04	0.000 ± 0.007

**Figure 2:**
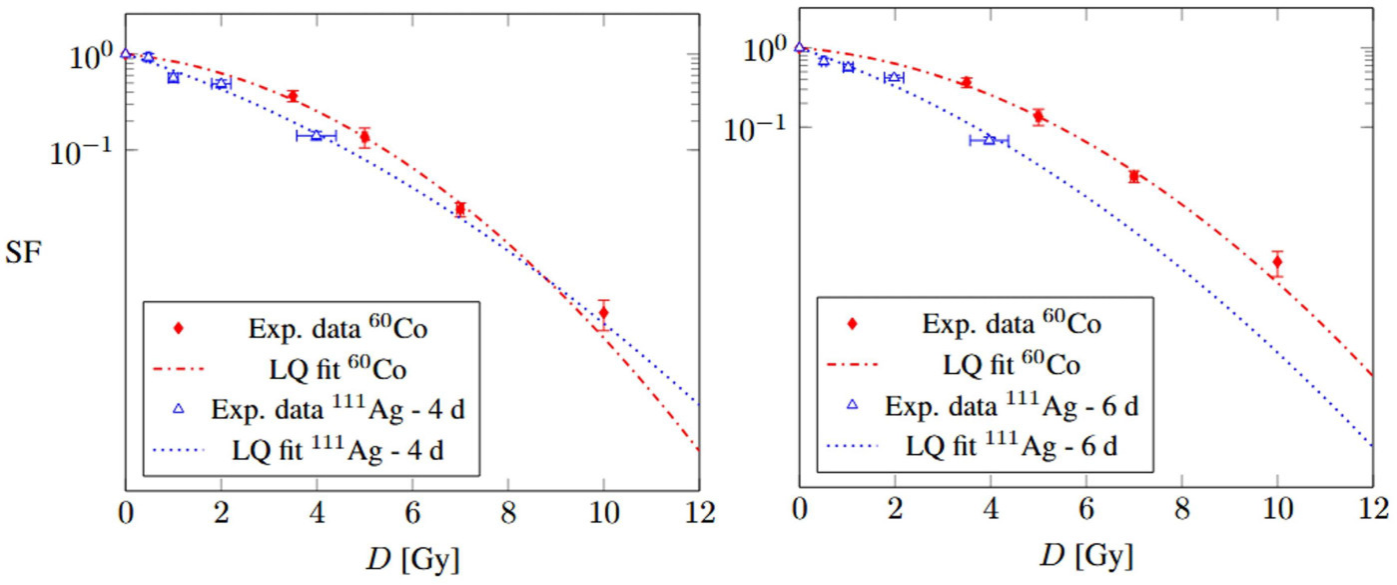
Cell survival curves of UMR-106 cells irradiated by ^60^Co and ^111^Ag after 4 d (left) and 6 d (right) of exposure.

Finally, the response of UMR-106 cells to ^111^Ag was compared to their response to ^60^Co, which had previously been measured as the reference curve for Boron Neutron Capture Therapy [15]. The ^60^Co photon irradiation of UMR-106 cells was previously performed at the San Matteo Polyclinic Foundation in Pavia, in a sealed camera, at a dose rate of 1 Gy/min. In this work, the response of UMR-106 cells to the γ radiation from ^60^Co was compared to that of ^111^Ag to assess the Relative Biological Effectiveness (RBE), considering ^60^Co as the reference radiation. RBE compares the severity of damage induced by a radiation source under test—delivering a dose $D_{\text{test}}$—relative to a reference radiation—delivering $D_{\text{ref}}$—for the same biological endpoint. The cell survival curves obtained with ^60^Co are compared to those resulting from ^111^Ag and fitted with the LQ model in Figure 2; the ^60^Co fit parameters are in Table 2. Based on the behavior of the curves, it was deemed appropriate to calculate the RBE only for the ^111^Ag radiation delivered over the 6-day period. In this case, the RBE at the endpoint of a 10% survival is 1.43. This RBE value, although depending on several radiobiological factors such as the cell line, the dose rate and the endpoint itself, is in line with those found in the literature for the $\beta^{-}$ radiation compared to the γ [26]. An RBE higher than 1 indicates that the studied radiation is more effective than the reference one in causing cell damage. This is expected as the radiobiological effect of ^111^Ag is due to electrons, generated close to sensitive cell sites, thus causing more damage than secondary electrons due to gamma external irradiation. Moreover, the dose rate could play a role in the radiobiological effectiveness.

**Table 2:** Linear-quadratic model parameters for the UMR-106 cell survival curves obtained for the exposure to ^60^Co (reference radiation for RBE evaluation).

Cell line	α [${\mathrm{Gy}}^{-1}$]	β [${\mathrm{Gy}}^{-2}$]
UMR-106	0.12±0.07	0.06±0.01

### 3.2 Foci analysis

**Figure 3:**
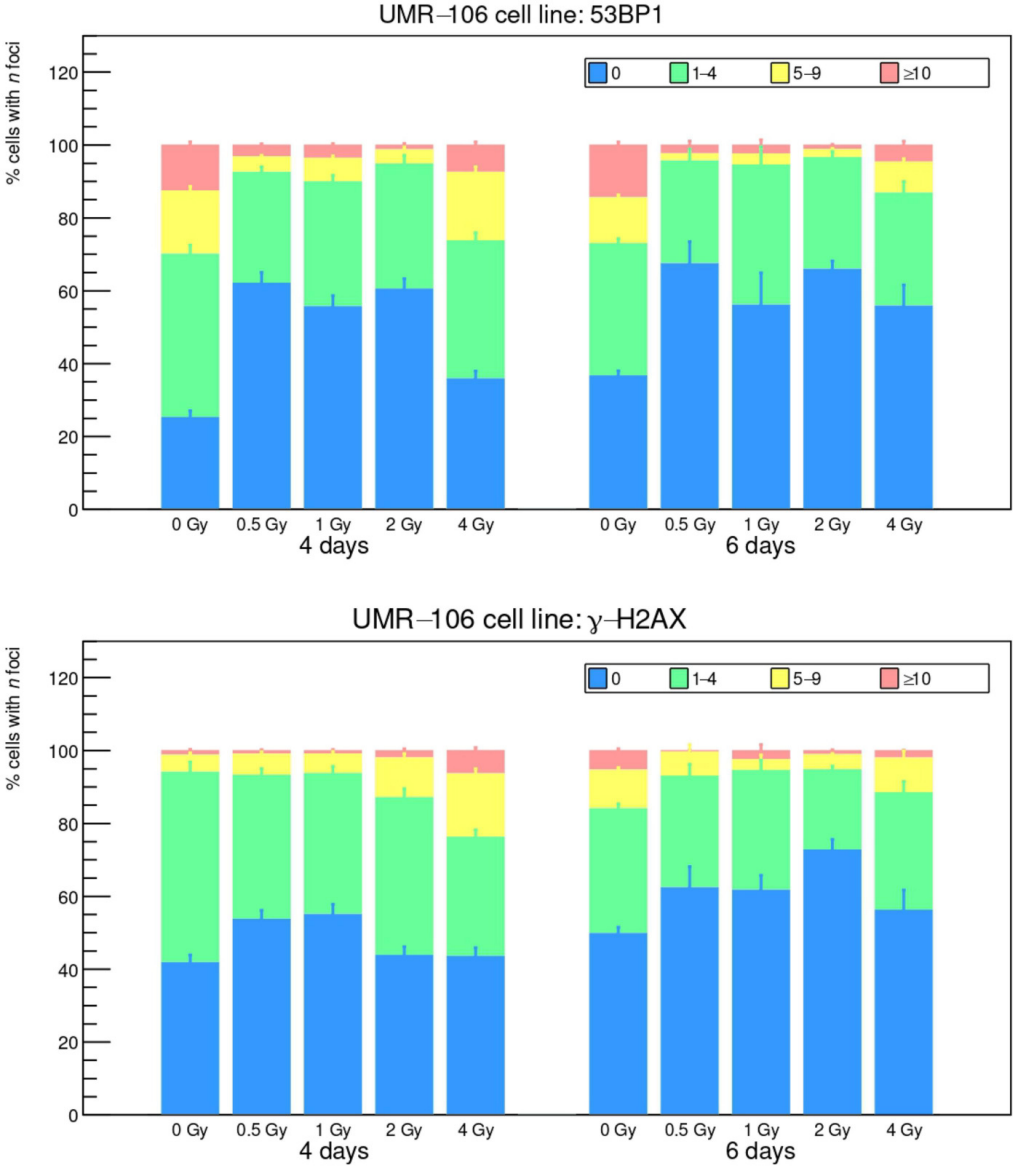
53BP1 and γ-H2AX *foci* induced by ^111^Ag decay radiation on the UMR-106 cell line after 4 and 6 d of exposure.

**Figure 4:**
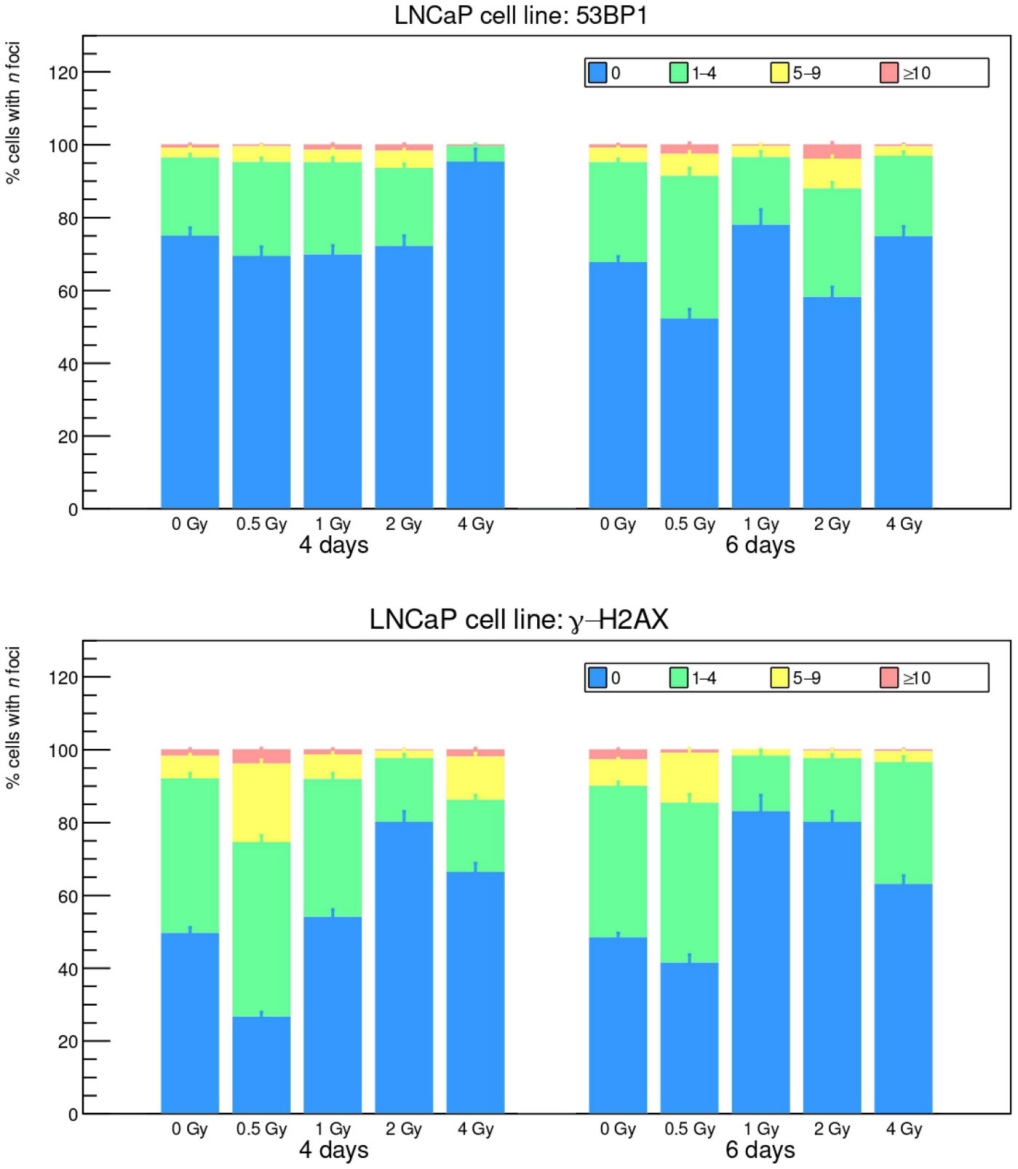
53BP1 and γ-H2AX *foci* induced by ^111^Ag decay radiation on LNCaP cell line after 4 and 6 d of exposure.

**Figure 5:**
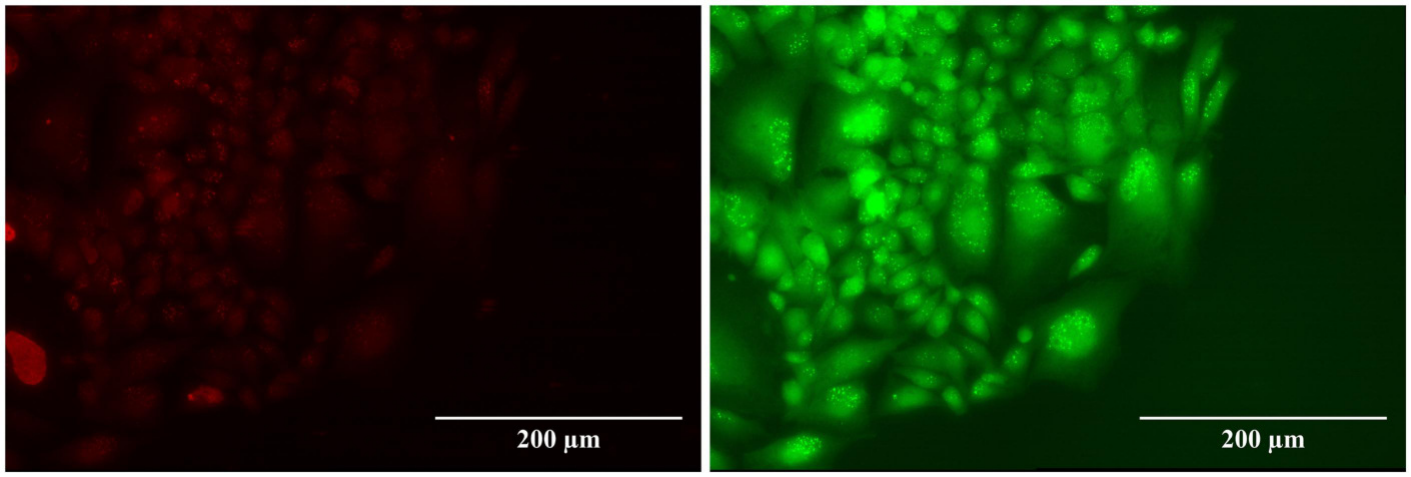
53BP1 *foci* (left) and γ-H2AX *foci* (right) of the UMR-106 cell line after 6 d of exposure to 4 Gy of ^111^Ag decay radiation.

The *foci* results, analyzed with the FIJI toolkit, are reported in Figures 3, 4, while a visual example is shown in Figure 5. The FIJI macro “Foci Analyzer” is used with its default intensity threshold for *foci* detection, set at three times the median standard deviation of the nuclear background signal in all nuclei, after difference-of-Gaussians background subtraction.

Cells are divided into four groups according to the recurrence of *foci* (0, 1–4, 5–9, ≥10). Long exposure times such as 4 and 6 d do not seem optimum for this kind of analysis, since the data for exposed cells look compatible with the control cultures (“0 Gy” bars), with the controls of a reference study [27] and, in general, with the known rate of natural DSB occurrence [28]. Furthermore, for both indicators 53BP1 and γ-H2AX, the control cultures show a higher presence of damage sites at 4 and 6 d: since control cells should only suffer from environmental DNA damage (or at maximum some low-intensity ^111^Ag γ rays from the other cultures in the incubator), this behavior may seem contradictory. A hypothesis can be that, at the end of the treatment, the dose rate is very low and the irradiated cells can repair DSBs much faster than the control thanks to the pathways triggered during the first days of exposure. In any case, it can be noted that LNCaP cells face a higher damage rate and are thus more sensitive to low activity concentrations compared to UMR-106 cells, in agreement with what we observed in the clonogenic assay.

### 3.3 Micronuclei analysis

**Figure 6:**
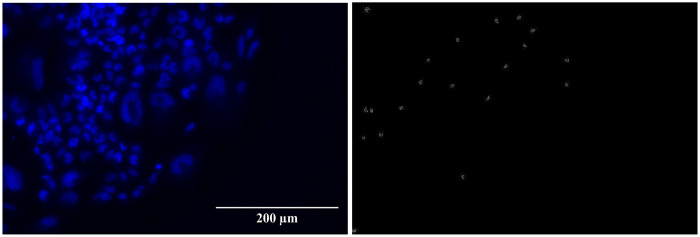
Cell nuclei colored with DAPI (left) and corresponding micronuclei detected with FIJI (right) of the UMR-106 cell line after 6 d of exposure to 4 Gy of ^111^Ag decay radiation.

**Figure 7:**
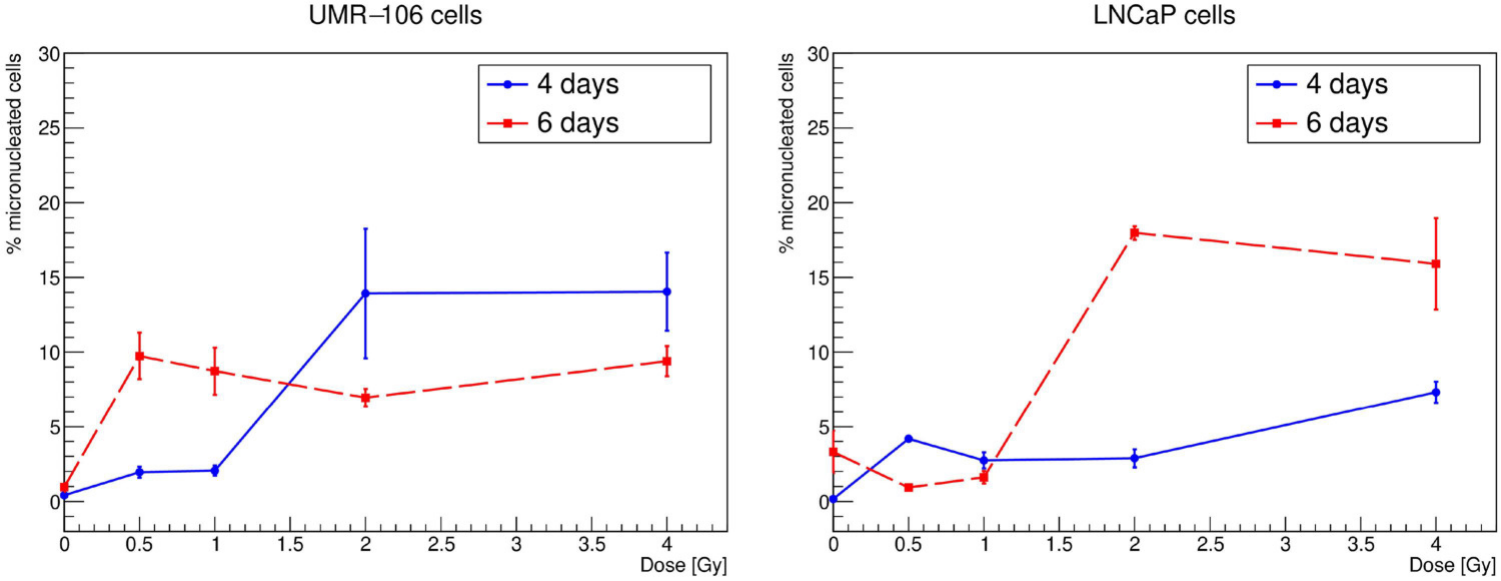
Micronuclei frequencies as a function of the absorbed dose for the UMR-106 (left) and LNCaP (right) cell lines after 4 and 6 d of irradiation.

Finally, the DAPI images can also be used for a simple procedure of micronuclei counting, carried out manually with the support of the FIJI toolkit (see Figure 6). The frequencies of cells exhibiting at least one micronucleus under the various conditions of initial activity and exposure time studied are shown in Figure 7. The uncertainty is computed as the standard error related to the number of available samples. Micronuclei can be expected to be much less sensitive to DNA repair dynamics than *foci*. As a matter of fact, the results, although with important fluctuations, generally associate a higher recurrence of micronuclei with higher absorbed doses: for both cell lines and both exposure times, frequencies are between 1% and 10% at 0.5–1 Gy and between 3% and 20% at 2–4 Gy.

## 4 Discussion

The present work is the first radiobiological study involving the radionuclide ^111^Ag, a candidate of interest for $\beta^{-}$ TRT. ^111^Ag production in nuclear reactors has been well documented in the literature, similarly to the biological procedures adopted in this investigation. Moreover, the concentrations considered have been proven not to impart any chemical toxicity to the cell cultures [16]. The experimental planning and subsequent dose-response analysis were guided by a previous cell dosimetry study based on Monte Carlo simulations with different codes [18] and by the well-known MIRD formalism [17]. Both radiobiological tests were applied to two cell lines, UMR-106 and LNCaP, and the absorbed-dose values were obtained under two different exposure times (4 and 6 d), reflecting different dose rates.

Generally speaking, the results of the clonogenic assay are in substantial agreement with similar experiments conducted with drugs loaded with other β-emitting radionuclides [29]. Whereas the two cell lines show overall similar sensitivity to the radioactive agent, the dose-rate variation produced opposite results: while LNCaP cells were found to be more sensitive to the higher dose rate, UMR-106 cells exhibited a lower survival curve for the lower dose rate. This difference may be evidence of higher dose-rate thresholds in certain UMR-106 DNA repair pathways, while LNCaP pathways operate more efficiently in the low-dose-rate regime. The RBE of ^111^Ag radiation with respect to ^60^Co γ emission at the endpoint of a 10% SF has also been calculated, giving a result >1 (as expected for a β emitter).

The data coming from the *foci* assay seem less significant than those from clonogenic survival, as a reduction in the *foci* with respect to the control cultures was observed. However, this outcome provides a hint about the long-term action of the triggered repair pathways. Moreover, this result will be important for the next experiments, since an upper limit to the exposure time has been set for studies aimed at analyzing the *foci*. On the other hand, the evaluation of the percentage of cells with micronuclei under each condition examined revealed a qualitative correlation with the absorbed dose, as expected. Looking at the results of all assays, the low number of *foci* in treated cells could also mean a low responsiveness to DNA damage, which could be correlated with a higher long-term recurrence of micronuclei and lower clonogenic survival. This hypothesis could be checked in further experiments by testing cell viability after treatment.

In future studies, such data could be useful in the development and tuning of biophysical models [30] that take into account the time evolution and population dynamics of cells treated with radionuclides. In addition, the opportunity to investigate which particular DNA DSB repair pathways among the known ones (NHEJ, Alt-NHEJ, HR, SSA) could have determined the different results for the two cell lines would be an enticing challenge from the biological point of view. Furthermore, this work will be a sort of “ice-breaker” for other radiobiological experiments investigating the therapeutic effects of ^111^Ag on other cancer cell lines and, hopefully, of the first ^111^Ag-labeled radiopharmaceuticals for TRT.

## Data Availability

The raw data supporting the conclusions of this article will be made available by the authors, without undue reservation.

## Footnotes

^1^The two S-values considered have different units since, in the reference [18], culture-medium S-values are normalized per activity concentration (and not activity) for easier experimental use.
